# CCN3: lactational bone booster

**DOI:** 10.1186/s13578-024-01344-z

**Published:** 2024-12-30

**Authors:** Nathan Xu, Kyle Yang, Mengjie Wang

**Affiliations:** 1 USDA/ARS Children’s Nutrition Research Center, Department of Pediatrics, Baylor College of Medicine, Houston, USA; 2 1100 Bates Street #8066, Houston, TX 77030 USA

**Keywords:** CCN3, ARH^ERα/Kiss1^ neurons, Bone formation, Lactation, Brain-breast-bone axis

## Abstract

Mammalian reproduction requires that nursing mothers transfer large amounts of calcium to their offspring through milk. Meeting this demand requires the activation of a brain-breast-bone circuit during lactation that coordinates changes in systemic hormones, dietary calcium intake, skeletal turnover, and calcium transport into milk. Classically, increased bone resorption via increased parathyroid hormone-related protein and low estrogen levels is the main source of calcium for milk production during lactation. Over the past few decades, investigators have described many aspects of this brain-breast-bone axis during lactation, yet many unanswered questions remain. Using a comprehensive set of parabiosis coupled with in vivo µCT, bone transplant studies, cell culturing and differentiation assays, mouse genetic models, pharmacologic interventions, hepatic viral transduction, and sequencing analysis, a recent study discovered that cellular communication network factor 3 (CCN3), derived from ARH^ERα/Kiss1^ neurons, functions as an osteogenic hormone to sustain bone formation and progeny survival during lactation. Compelling evidence has been presented to show that (1) CCN3 expression in ARH^ERα/Kiss1^ neurons fluctuates, almost exclusively appearing during lactation; (2) CCN3 stimulates mouse and human skeletal stem cell activity, increases bone remodeling and fracture repair in young and old mice of both sexes; (3) knockdown *Ccn3* transcripts in the ARH^Kiss1^ neurons in lactating dams causes devastating bone loss and failure to sustain progeny survival. These findings suggested that the stage-specific expression of CCN3 in female ARH^ERα/Kiss1^ neurons during lactation is a newly identified brain-bone axis evolved to sustain the skeleton in mammalian mothers and offspring.

## Main text

**Fig. 1:**
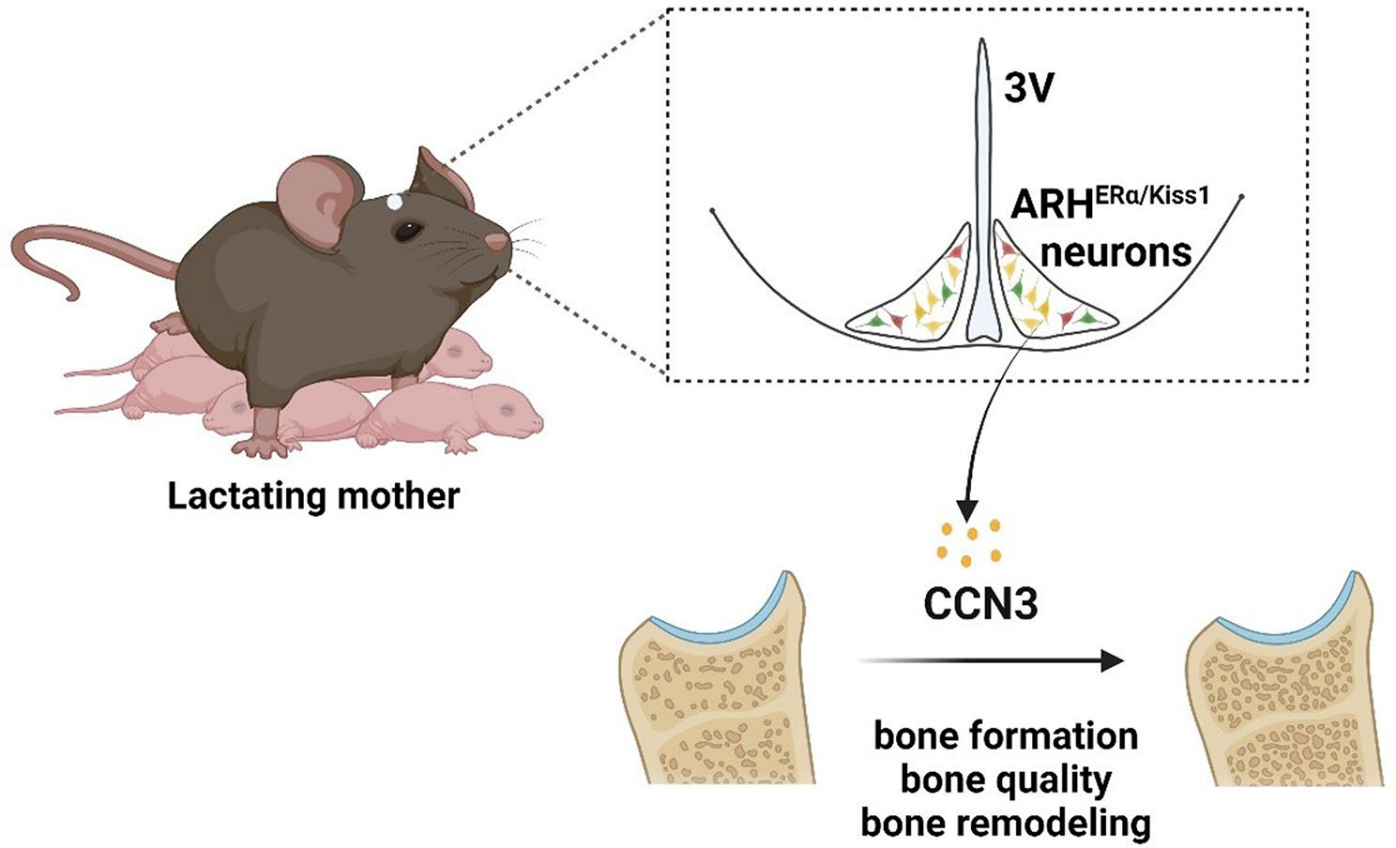
CCN3, derived from ARH^ERα/Kiss1^ neurons, functions as an osteogenic hormone to sustain bone formation and progeny survival during lactation.*3V* third ventricle, *ARH* arcuate nucleus of the hypothalamus, *CCN3* cellular communication network factor 3, *ERα* estrogen receptor α, *Kiss1* kisspeptin

The neonatal period represents the most rapid period of skeletal growth; thus, milk must supply large amounts of calcium. Suckling stimulation suppresses the number of kisspeptin (Kiss1) neurons and subsequent gonadotropin-releasing hormone secretion, which results in hypothalamic hypogonadism and low estrogen levels [6]. As a result, the low estrogen and increased parathyroid hormone-related protein levels trigger bone resorption, which liberates calcium from the skeleton into the circulation and promotes calcium transport into milk [7]. It has been comprehensively documented in different species that markedly increased bone resorption leads to lactation-associated bone loss. Women typically lose between 5% and 10% of bone mineral content within 3–6 months of exclusive breastfeeding [1], while female mice lose between 20% and 33% of bone mass compared to age-matched nulliparous controls [9]. Fortunately, lactating women [2] and mice [9] gain bone rapidly after cessation of lactation and post-weaning. However, in some rare but severe cases, women can develop pregnancy- and lactation-associated osteoporosis (PLO) and suffer vertebral compression fractures and back pain during the third trimester of pregnancy or early postpartum [8]. Despite extensive investigations, knowledge in this field remains limited, which motivates further scientific studies. Babey and colleagues used a combination of question-driven and discovery-based approaches and identified brain-derived cellular communication network factor 3 (CCN3) as a potentially new therapeutic osteoanabolic hormone that defines a novel female-specific brain-bone axis for ensuring mammalian species survival (Fig. 1).

Previous studies from the same group [3, 5] have shown that central estrogen signaling exerts a sex-dependent restraint on bone formation. Given the cellularity complexity of estrogen receptor α (ERα) neurons in the medial basal hypothalamus (MBH), including the arcuate nucleus (ARH) and the ventrolateral subdivision of the ventromedial hypothalamic nucleus (vlVMH), the authors leveraged the *Esr1*^*Nkx2 − 1Cre*^ mouse model, in which MBH ERα is eliminated via *Nkx2-1*-driven Cre recombinase [3]. Surprisingly, *Esr1*^*Nkx2 − 1Cre*^ females exhibited a remarkably high bone mass phenotype by four weeks of age [5]. The ARH^Kiss1^ neurons were further identified as the critical estrogen-responsive subpopulation for promoting this robust bone mass in female mice [5]. Given the privileged position of the ARH that lies dorsal to the median eminence, a circumventricular organ of the brain, the authors questioned if the high bone mass in *Esr1*^*Nkx2 − 1Cre*^ females originates from a circulatory factor.

The authors performed the parabiosis coupled with in vivo µCT imaging by surgically joining two female groups, generating *Esr1*^*fl/fl*^ (WT) with WT or *Esr1*^*Nkx2 − 1Cre*^ (Mut) pairs. Unlike the females in the WT-WT pairing, the *Esr1*^*fl/fl*^ and *Esr1*^*Nkx2 − 1Cre*^ females in the WT-Mut pairing gained significantly higher bone mass with higher fractional bone volume [percent bone volume/total volume (%BV/TV)]. The comparable uterine weights and other metabolic parameters between both pairings imply that the high bone mass in *Esr1*^*Nkx2 − 1Cre*^ females was not seemingly modulated by estrogen. To further explore the existence of the osteoanabolic factor that accounts for the high bone mass in *Esr1*^*Nkx2 − 1Cre*^ females, the authors performed bone transplant studies in which female and male femurs from 4-week-old *Esr1*^*fl/fl*^ donors were implanted subcutaneously into 8-week-old *Esr1*^*fl/fl*^ or *Esr1*^*Nkx2 − 1Cre*^ females. Interestingly, significant increases in %BV/TV were detected in *Esr1*^*Nkx2 − 1Cre*^ females after implantation with femurs from female and male mice, indicating that this female-specific osteoanabolic factor functions in both sexes.

Previous studies demonstrated that osteochondral skeletal stem cells (ocSSCs) facilitate new bone formation by forming bone and cartilage [4]. To examine whether the brain-derived osteoanabolic factor boosts the activity of ocSSC, the authors transplanted WT ocSSCs into both WT and *Esr1*^*Nkx2 − 1Cre*^ mice. WT ocSSCs grafted into the *Esr1*^*Nkx2 − 1Cre*^ females demonstrated significantly higher levels of mineralization compared to controls, suggesting that the osteoanabolic factor in *Esr1*^*Nkx2 − 1Cre*^ females promotes bone growth via alteration of the ocSSC lineage. Consistently, the higher bone mass observed in WT-Mut parabionts or WT bone transplants correlated with increased ocSSC frequency. To further verify the potency of this osteoanabolic factor, GPF-positive WT ocSSCs were stereotaxically delivered into the MBH, where the ARH is located. µCT imaging revealed mineralized ossicles overlapping with the GFP-positive cells in *Esr1*^*Nkx2 − 1Cre*^ mice but not in control mice six weeks after the injection, providing further evidence of a circulatory variable that likely originates from the ARH or surrounding regions. Using flow cytometric analysis and differentiation assays, Babey and colleagues found a sex-dependent increase in ocSSC frequency in 3- and 10-week-old *Esr1*^*Nkx2 − 1Cre*^ females. Additionally, *Esr1*^*Nkx2 − 1Cre*^ ocSSCs exhibited a higher potential for bone and cartilage formation, including cells harvested from 54-week-old females, supporting that these *Esr1*^*Nkx2 − 1Cre*^ ocSSCs can prevent bone loss across a wide age range. Along with the remarkable neural stem cell injection, the authors effectively discussed how the circulatory factor building bone formation originates from the ARH surrounding area.

Single-cell RNA sequencing data implied that *Esr1*^*Nkx2 − 1Cre*^ ocSSCs differentiation dynamics were primed toward bone formation, yet few hints were detected regarding the identity of the osteoanabolic hormone in *Esr1*^*Nkx2 − 1Cre*^ females. Given the key role of the ARH in the control of energy balance [10], the authors hypothesized that a chronic high-fat diet (HFD) challenge might influence ARH function in *Esr1*^*Nkx2 − 1Cre*^ females and potentially reveal gene candidates responsible for the sex-dependent brain-bone axis. Notably, the HFD reversed the high bone mass and bone strength phenotype in *Esr1*^*Nkx2 − 1Cre*^ females with unaltered bone resorption and metabolic parameters. No effects were seen in *Esr1*^*Nkx2 − 1Cre*^ males. Regardless of the degradation of dense bones, *Esr1*^*Nkx2 − 1Cre*^ females unexpectedly resisted fat accumulation in the tibia as quantified by osmium staining, which defied the currently known coupling between bone mass adipose tissue expansion and bone loss.

Then, the authors successfully captured the genetic changes associated with the dietary-induced loss of bone mass in *Esr1*^*Nkx2 − 1Cre*^ females by profiling microdissected ARH tissues. Bulk RNA-seq of the ARH revealed a small set of upregulated differentially expressed genes encoding neuropeptides or secreted proteins, including *Ccn3*, *Fst*, *Grp*, and *Penk*, which all dropped significantly after HFD in females. Specifically, *Ccn3* and *Penk* expression by RNAscope were consistently found in the basal region of non-overlapping neurons in the ARH of *Esr1*^*Nkx2 − 1Cre*^ females and disappeared after the HFD challenge. No other candidates emerged after profiling the pituitary and liver, the other common tissue sources of secreted proteins, except the pituitary *Penk*. Notably, *Ccn3*, not *Penk*, highly overlapped with ARH^Kiss1^ neurons in *Esr1*^*Nkx2 − 1Cre*^ females. Thus, the cells with high expression of CCN3 in the ARH of *Esr1*^*Nkx2 − 1Cre*^ females are Kiss1-positive and ERα-negative, bridging the connections among CCN3, ARH^ERα/Kiss1^ neurons, and bone turnover. Importantly, the authors provided compelling evidence that CCN3 is a promising candidate for coordinating the brain-breast-bone axis.

The current understanding of CCN3 in bone turnover remains controversial. The authors conducted a series of experiments to verify the function of CCN3. They performed osteogenic differentiation assays of purified primary mice ocSSC treated daily with the recombinant mice (m)CCN3 protein and found that bone mineralization increased by 200%. Peptides encoded by *Penk* failed to produce any change. This experiment was repeated on primary human ocSSCs treated with recombinant human (h)CCN3 protein. Consistently, the lower dose (0.025–0.25 nM) of CCN3 effectively promoted osteogenesis in both mouse and human ocSSCs. Next, the authors found that female and male femurs when treated with plasma isolated from *Esr1*^*Nkx2 − 1Cre*^ females had significantly higher bone mass than the contralateral femurs treated with plasma from WT female and male. Daily treatment of mCCN3 (3 nM) for 5 days induced similar changes of increased %BV/TV compared with untreated baseline contralateral female and male femurs. In addition, the in vivo findings showed that daily injection of mCCN3 (7.5 µg/kg, i.p.) for 21 days significantly increased bone mass compared to saline in adult wild-type mice of both sexes. Notably, in a stabilized fracture model of 2-year-old male mice, callus %BV/TV and strength exhibited dose-dependent increases, suggesting mCCN3 improves fracture repair in males. Taken together, Babey and colleagues provided ex vivo and in vivo findings to address that the lower dose of CCN3 effectively promoted osteogenesis in both mice and humans. Interestingly, they observed the inhibitory effects in both mouse and human ocSSC differentiation assays at higher CCN3 doses. To fully understand the multifaceted functions of CCN3 in bone formation, future studies are needed to identify the molecular target of CCN3 in ocSSCs and possibly other cellular populations, including osteocytes that reversibly remodel the perilacunar and canalicular matrix during lactation.

To establish the linkage between CCN3 and increased bone mass in *Esr1*^*Nkx2 − 1Cre*^ females, the authors carried out the in vivo CCN3 gain-of-function and loss-of-function experiments. The transient knockdown of *Ccn3* expression in the ARH of *Esr1*^*Nkx2 − 1Cre*^ females via stereotaxic injection of *Ccn3-*siRNA profoundly attenuated the dense bone phenotype with levels of *Ccn3*/CCN3 closely tracking %BV/TV. These findings highlight that the high bone mass in *Esr1*^*Nkx2 − 1Cre*^ females relies on brain CCN3. Given the secretary capacity of hepatocytes, ectopic expression of mCCN3 in hepatocytes was detected in WT females and males following retroorbital injection of AAV-dj-CCN3. Plasma CCN3 detection only happened after heparin-agarose purification from the highest levels of hepatic CCN3 expression, demonstrating the poor specificity of existing anti-CCN3 antibodies. The potency of CCN3 as an osteoanabolic hormone was further established by a %BV/TV rise after expressing CCN3 in the 5-month-old ovariectomized WT females and the 20-23-month-old WT females. Importantly, CCN3 increased bone formation parameters such as bone formation rate per bone surface and number of osteoblasts per bone surface but did not change bone resorption-related parameters such as number of osteoclasts per bone surface and lacunar density per bone area. This result implies that CCN3 also promotes healthy bone remodeling in both sexes.

To examine the role of CCN3 in the brain-breast-bone axis during lactation, the authors examined the dynamic ARH CCN3 expression during pregnancy, lactation, and different postpartum stages. Like virgin intact females, CCN3 expression in ARH^ERα^ neurons was non-existent in the early and late stages of pregnancy. Surprisingly, by seven days postpartum, CCN3 was abundantly expressed in ARH^ERα/Kiss1^ neurons in the lactating dams, reaching near equivalent levels as in *Esr1*^*Nkx2 − 1Cre*^ females. Additionally, the forced weaning decreased CCN3 in ARH^ERα^ neurons when examined 3 or 7 days after the removal of pups. This experiment underscored that CCN3 expression in ARH^ERα/Kiss1^ neurons almost exclusively appears during lactation. One future direction is how *Ccn3* expression is triggered in the ARH in females. Unlike the known markedly increase in prolactin signaling during lactation, levels of CCN3 only modestly increased in mutant female mice [5], suggesting that alternative mechanisms may involve calcium sensing during lactation. However, CCN3 was absent in the ARH of ovariectomized WT females, suggesting estrogen depletion alone is insufficient to induce CCN3 production in ARH^ERα/Kiss1^ neurons. This dynamic change of CCN3 expression in ARH^ERα/Kiss1^ neurons and accompanied high bone mass fill the void of estrogen (with rapid drop after delivery) to counteract excessive bone loss during lactation.

Lastly, to confirm that CCN3 is an anabolic brain hormone during lactation, the authors delivered the viral vectors carrying short hairpin RNA (shRNA) targeting *Ccn3* (sh*Ccn3*) to knockdown CCN3 in the ARH in adult virgin females before pregnancy. sh*Ccn3* dams had comparable fertility, fecundity, and milk provision, but experienced a 31% reduction in bone mass when fed calcium-rich breeder chow (0.8% Ca^2+^). When sh*Ccn3* dams were challenged with a low-calcium diet (0.01% Ca^2+^) postpartum, their pups had significantly lower body weight than controls, suggesting the role of brain CCN3 in facilitating inter-generational resource transfer. 4 out of 6 pups nursed by one sh*Ccn3* dam failed to thrive, leading to increased mortality. In addition, opposite to the 30% weight gain in pups nursed by control dams, 10% weight loss occurred in pups transferred to sh*Ccn3* dams, suggesting the pup viability depends on the status of brain CCN3 in dams. Thus, there is convincing evidence that ARH^ERα/Kiss1^ neurons produce CCN3 during lactation to maintain the maternal skeleton integrity and viability of offspring.

In summary, Baby and colleagues discovered that ARH^ERα/Kiss1^ neurons-derived CCN3 functions as an osteoanabolic hormone that sustains bone in lactating females by lifting the restraints on bone formation, providing a novel mechanism underlying the coordination of the brain-breast-bone axis during lactation. Their findings also established that CCN3 is a potentially new therapeutic osteoanabolic hormone for both sexes and defined a new maternal brain hormone for ensuring species survival in mammals. Yet it remains unclear what signals the increased expression of CCN3, with prolactin signaling being a possibility. Future directions of research include the potential translation of the novel CCN3 findings in genetic and chronic bone diseases.

## Data Availability

Not applicable.

## Abbreviations

ARH: Arcuate nucleus of the hypothalamus
%BV/TV: Percentage bone volume/total volume
CCN3: Cellular communication network factor 3
ERα: Estrogen receptor α
HFD: High-fat diet
Kiss1: Kisspeptin
MBH: Medial basal hypothalamus
ocSSC: Osteochondral skeletal stem cells
PLO: Pregnancy- and lactation-associated osteoporosis
shRNA: Short hairpin RNA
vlVMH: Ventrolateral subdivision of the ventromedial hypothalamic nucleus
WT: Wild-type
